# A20 as a Potential Therapeutic Target for COVID‐19

**DOI:** 10.1002/iid3.70127

**Published:** 2025-01-24

**Authors:** Yongyao Wu, Lilan He, Rong Li, Jiuxuan Li, Qing Zhao, Bin Shao

**Affiliations:** ^1^ State Key Laboratory of Oral Diseases and National Clinical Research Center for Oral Diseases, West China Hospital of Stomatology Sichuan University Chengdu China; ^2^ Laboratory of Radiation Biology, Laboratory Medicine Centre, Department of Blood Transfusion The Second Affiliated Hospital Army Military Medical University Chongqing China

**Keywords:** A20, COVID‐19, immunosuppression, inflammatory response, STING

## Abstract

**Background:**

Coronavirus disease 2019 (COVID‐19), caused by the severe acute respiratory syndrome coronavirus 2 (SARS‐CoV‐2), is a major concern due to its astonishing prevalence and high fatality rate, especially among elderly people. Patients suffering from COVID‐19 may exhibit immunosuppression in the initial stage of infection, while a cytokine storm can occur when the disease progresses to a severe stage. This inopportune immune rhythm not only makes patients more susceptible to the virus but also leads to numerous complications resulting from the excessive production of inflammatory factors. A20, which is widely accepted as a pivotal regulator of inflammation, has been shown to be implicated in the processes of antiviral responses and immunosuppression. Thus, A20 may participate in regulating the pathological processes of COVID‐19.

**Methods:**

This narrative literature review summarizes recent evidence on the mechanisms of A20 in regulating the pathological processes of COVID‐19. We also downloaded single‐cell RNA‐seq data sets from healthy individuals and patients with varying severities of COVID‐19 from the NCBI GEO database to further dissect A20's regulatory mechanisms of these intricate cytokine pathways that are closely associated with SARS‐CoV‐2 infection.

**Results:**

A20 might be one of the most critical anti‐infectious and anti‐inflammatory factors involved in the pathogenesis of COVID‐19. It effectively suppresses the immune damage and inflammatory storm caused by viral infection.

**Conclusions:**

Understanding the relationship between A20‐regulated signaling pathways and pathological processes of COVID‐19 can provide insight into potential targets for intervention. Precise regulation of A20 to induce antiviral activity and an anti‐inflammatory response could mediate the pathogenesis of COVID‐19 and could become an effective treatment.

## Graphical abstract

Coronavirus disease 2019 (COVID‐19) is characterized by abnormal immunosuppression in the incipient stage and drastic cytokine storm in the severe stage. A20 (TNFAIP3) is proved to mediate antiviral response through impact on the RIG‐I pathway and STING pathway, while dampening cytotoxic T cell responses in bronchial epithelial cells. By analyzing the RNA‐seq data set, we identified some classic proinflammatory cytokines and and found that their expression varies significantly among healthy individuals and patients with different severities of viral infections.

## 1 Introduction

Coronavirus disease 2019 (COVID‐19) has caught global attention as a public health emergency of international concern [1, 2], which is caused by severe acute respiratory syndrome coronavirus 2 (SARS‐CoV‐2) and first came to people's attention in December 2019 [3]. The SARS‐CoV‐2 consists of a positive‐sense single‐stranded RNA genome and various structural proteins (NSPs). The viral genome mainly encodes two large open reading frames (ORFs), ORF1a and ORF1b, as well as several smaller ORFs. The NSPs of SARS‐CoV‐2 include spike (S), nucleocapsid (N), envelope (E), membrane (M) proteins, and accessory proteins [4]. Symptoms of COVID‐19 can range from fever and dry cough to complications of multiple organ failure, acute respiratory distress syndrome (ARDS), and shock [5, 6], which has seriously threatened the physical health and quality of life of human beings. Moreover, it has spread globally with sustained human‐to‐human transmission among close contacts [7]. SARS‐CoV‐2 infection can cause detrimental tissue damage by reason of excessive inflammatory innate responses and stunted adaptive immune responses [8]. There is also evidence that suggests that reducing the virus burden drives inflammation that requires correction, thus ameliorating adverse outcomes [9]. However, the treatment using corticosteroids is highly controversial as they are accused of exacerbating COVID‐19‐associated lung injury [5, 10]. Additionally, JAMA has reported that the COVID‐19 fatality rate has reached up to 30.49% among patients aged 85 years or older in the United States [11]. COVID‐19 has become the norm. To evade the attack of the immune system and better infect the host, SARS‐CoV‐2 has undergone multiple mutations, leading to the inevitable reinfection of the body [12]. Over time, new virus variants may become less pathogenic, but more infectious [13].

Research indicates that the 3′ end of the SARS‐CoV‐2 genome contains ORFs that encode the S, M, E, and N proteins, which are essential for the virus's function and structure [14, 15]. Angiotensin‐converting enzyme 2 (ACE2) is the key receptor for SARS‐CoV‐2 infection of host cells. S protein first binds to the ACE2 receptor on the host cell, initiating the infection process after entering the cell [16, 17, 18, 19], and triggers an immune‐inflammatory response within the body, such as activating toll‐like receptors (TLR), leading to the release of proinflammatory cytokines [20]. The E protein regulates cell lysis and the release of the viral genomic material and can also activate the E protein NOD‐like receptor family, pyrin domain containing 3 (NLRP3) inflammasome [15, 21]. The N protein is essential for the replication and packaging of viral RNA. Additionally, it interacts directly with the NLRP3 inflammasome, facilitating its assembly and activation. This interaction enhances the function of the NLRP3 inflammasome and triggers the Nuclear Factor kappa‐light‐chain‐enhancer of activated B cells (NF‐κB) signaling pathway, which plays a crucial role in mediating inflammatory responses [15, 21, 22].

A20 (encoded by *TNFAIP3*) is widely accepted as a pivotal protein in regulating inflammation. It has the ability to restrict several pathways involved in the pathological processes of inflammation, such as dampening the secretion of Tumor Necrosis Factor (TNF), Interleukin‐6 (IL‐6), and other cytokines, as well as downregulating the quantity and function of inflammasomes like NLRP3, which is recognized as a potential pathogenic mechanism of COVID‐19 [23, 24, 25]. Additionally, the overactivation of the immune response is related to the poor prognosis of clinical treatment in COVID‐19 [25]. Furthermore, A20 can function as a regulator of cell death pathways such as necroptosis and pyroptosis, thereby reinforcing its ability to restrict the production of proinflammatory cytokines [26]. Additionally, A20 also possesses the property to control the activation of dendritic cells (DCs) and DC‐mediated T‐cell stimulation, thereby imposing restrictions on excessive systemic immune responses [27, 28].

A20, as an anti‐inflammatory protein, has been demonstrated to have crucial properties in suppressing NF‐κB activation [29], while NF‐κB is implicated in the processes of antiviral response through the retinoic acid‐inducible gene I (RIG‐I) pathway and the TLR pathway [30, 31]. Thus, A20 has demonstrated its role in resistance during the antiviral response. In the NF‐κB pathway, NF‐κB inhibitors, such as A20, transform infectious signals into oscillatory or pulse‐like responses, which prevents excessive inflammation but also imposes restrictions on the antiviral state of cells [32]. Furthermore, A20 has been shown to suppress interferon (INF) regulatory factor 3 (IRF3)‐mediated innate antiviral responses, resulting in the downregulation of IFN and IFN‐stimulated genes (ISGs) and facilitating viral replication in influenza A virus (IAV) infection [33, 34]. Surprisingly, airway epithelial cell‐specific A20 knockout mice (A20^AEC‐KO^) exhibit dampened immune response and lung damage during IAV infection compared to their wild‐type littermates, which may be due to attenuated cytotoxic T cell (CTL) responses [35]. Additionally, A20 might serve as an important ubiquitin editing enzyme in regulating the activity of the stimulator of IFN genes (STING), thus impairing type I IFN production [36, 37]. These pieces of evidence suggest that A20 might contribute to hindering the antiviral response in SARS‐CoV‐2 infection and exacerbating respiratory symptoms, further indicating that A20 could be a potential therapeutic target in COVID‐19.

In this review, we sum up the mechanism of A20 in interrupting antiviral response and restricting inflammation, especially emphasizing its role in the respiratory system, thereby providing ideas for the clinical treatment of COVID‐19. We also highlight this review by analyzing the single‐cell RNA‐seq data set of healthy individuals and patients with varying severity of COVID‐19 disease obtained from the NCBI GEO database, deeply dissecting regulatory mechanisms of intricate cytokines pathways that are closely associated with SARS‐CoV‐2 infection, hoping to corroborate the hypothesis we described and cast a light on A20‐releted antiviral research.

## 2 Clinical Symptoms and Inflammatory Characters of COVID‐19

Coronaviruses are of high concern because of their high prevalence and wide distribution and the possibility of emerging periodically [38]. SARS‐CoV‐2 is a clade genetically different from Ber coronaviruses such as SARS‐CoV and Middle East respiratory syndrome coronavirus (MERS‐CoV), which have also caused pandemics [6]. There is evidence demonstrating that SARS‐CoV‐2 possesses superior plasma membrane fusion ability and higher infectivity compared to SARS‐CoV and MERS‐CoV [39], but COVID‐19 had a lower fatality rate than both SARS and MERS (10% for SARS‐CoV and 37% for MERS‐CoV) [40, 41]. Epidemiologic research revealed that the susceptibility and fatality risks of SARS‐CoV‐2 infection were considerably age‐dependent [41, 42]. In addition, SARS‐CoV‐2 has been tested as a zoonotic virus, and even cats that have close contact with humans are permissive to infection and susceptible to airborne infection [43].

Chest CT imaging abnormalities have been observed in COVID‐19 pneumonia, and asymptomatic patients have also shown a rapid evolution from focal unilateral to diffuse bilateral changes on CT scans [41]. And in the SARS‐CoV‐2‐infected macaque model, the virus was detected in ciliated epithelial cells of nasal, bronchial, and bronchiolar mucosae, accompanied by diffuse alveolar damage of type I and II pneumocytes [44]. Moreover, virological analysis offered evidence for active replication of SARS‐CoV‐2 in upper respiratory tract tissues and lung tissues [45, 46]. Surprisingly, even though SARS‐CoV‐2 is more efficient at replicating than SARS‐CoV, it significantly failed to induce IFNs I, II, or III in infected human lung tissues. Furthermore, patients with severe COVID‐19 have been found to have decreased IFN‐γ expression in CD4^+^ T cells [47, 48]. Studies have found that SARS‐CoV‐2 NSP1, NSP2, NSP5, NSP6, NSP8, NSP15, M, ORF3b, ORF6, ORF7b, ORF9b, and ORF10 all inhibit IFN response [49, 50, 51, 52, 53, 54, 55, 56]. In addition, nearly all COVID‐19 patients presented lymphopenia, which manifested as decreased numbers of T lymphocytes in vivo, particularly CD4^+^ and CD8^+^ T cells, and resulted in further reduction in IFN‐γ production [57]. All of these factors might be responsible for the failure to resist viral infections, even though the precise mechanism of immunosuppression remains unrevealed.

COVID‐19 is also characterized by a cytokine storm, and patients with severe COVID‐19 exhibit drastically elevated levels of proinflammatory cytokines such as IL‐6, IL‐1 beta (IL‐1β), IL‐17, and TNF‐α [58]. Cytokine storms may lead to extensive pulmonary pathology and numerous complications, such as shock and tissue damage in vital organs, as well as multiple organ failure [8, 47]. For instance, systemically elevated cytokines can be cardiotoxic, thus giving rise to myocardial injury, and they also often cause fatal damage by reducing blood flow to the kidneys. Moreover, significant inflammatory response can result in disseminated intravascular coagulopathy (DIC) in severe patients and can excessively promote blood clotting, then potentially trigger strokes [59, 60]. Levels of these cytokines are closely associated with the prognosis in patients with severe COVID‐19, and it makes sense that IL‐6 has been identified as a stable indicator of poor outcomes [5]. Correspondingly, the IL‐6 receptor blockade, named tocilizumab, has been approved for use in patients with COVID‐19 who exhibit cytokine release syndrome [5]; the trial of using IL‐1 blockade (anakinra) in sepsis has been applied in clinical treatment as well [61].

## 3 Mechanism of A20 Regulating Antiviral Response

### 3.1 A20 Regulates Antiviral Response Through Impact on the RIG‐I Pathway

**Figure 1:**
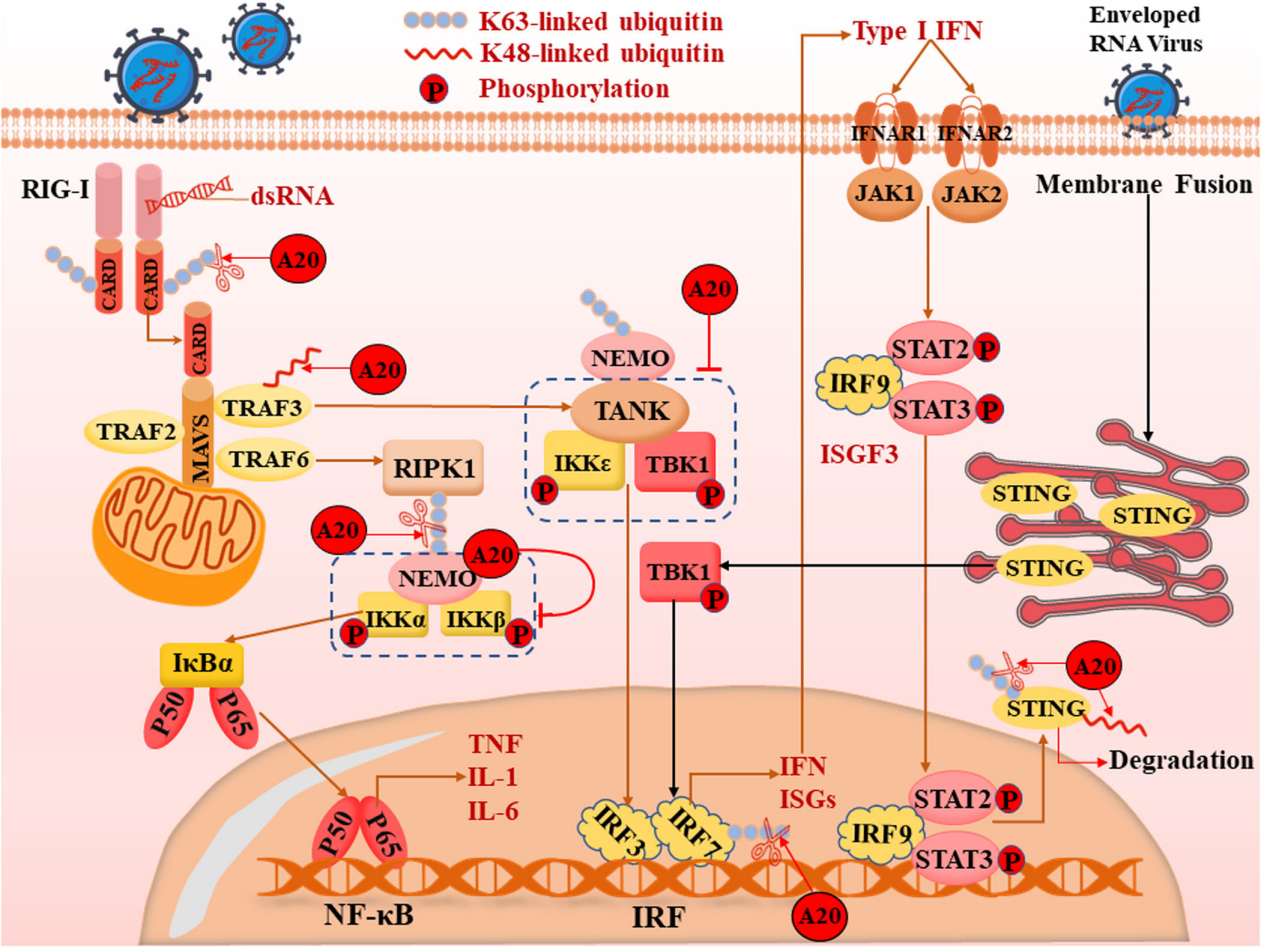
Mechanism by which A20 regulates antiviral response. In the RIG‐I pathway, A20, as a ubiquitin editing enzyme, has the capability to impair IFN response relying on RIG‐I in many ways. Additionally, A20 has the ability to interact with TBKI and IKKε to inhibit their activity, thus blocking phosphorylation and subsequent dimerization of IRF3. Moreover, A20 can impair IRF7 transcriptional activity by negatively regulating the K63‐linked polyubiquitination of IRF7 through its N‐terminal OTU domain. NF‐κB activation can be interrupted by A20, resulting in the production of antiviral cytokines. First, A20 could remove K63‐linked ubiquitin chains from RIPK1 and add K48‐linked ubiquitin on RIPK1, thus targeting RIPK1 for proteasomal degradation and ultimately preventing the activation of NF‐κB and protecting the cells. Second, A20 can also inhibit the IKK complex activity via its recruitment to the NEMO C‐terminus, thus destabilizing the NF‐κB activation. Activity and stability of STING are dependent on various ubiquitin modifications. A20 may be an important protein targeting STING and degradation of STING through ubiquitin editing may be mediated by A20. This potential role of A20 in regulating STING blocks the activation of IRF3, thus influencing the subsequent production of IFN.

RIG‐I is a human pattern recognition receptor (PRR) that can sense the 5′‐triphosphorylated double‐stranded RNA (dsRNA) produced during viral replication, and its activation triggers an immune response [62]. Loske et al. reported higher basal expression of relevant PRRs, such as RIG‐I (ddx 58) and Melanoma Differentiation‐Associated Protein 5 (MDa5) (ifih 1), in innate immune cells of children's upper airways. Therefore, children infected with SARS‐CoV‐2 show a stronger innate antiviral response [63]. Caspase activation and recruitment domains (CARDs) are located in the N terminus of RIG‐I (RIG‐I‐N), and CARD–CARD interactions can mediate the association of K63‐linked polyubiquitin chains between RIG‐I and mitochondrial antiviral signaling (MAVS), thereby resulting in the transmission of upstream signaling to the MAVS protein [64, 65]. In this process, K63‐linked ubiquitination on the N terminus of RIG‐I (RIG‐I–N), which is dependent on Tripartite Motif Containing 25 (TRIM25), is essential because it is vital to the activity and stability of MAVS [64, 66]. What is remarkable is that nonstructural protein 1 (NS1) of IAV can target TRIM25 to block downstream signaling, thereby preventing an efficient host immune response [67]. When RIG‐I is ubiquitinated and MAVS is converted into a prion‐like polymer, MAVS's TNF Receptor‐Associated Factor (TRAF)‐interacting motifs (TIMs) of MAVS can recruit ubiquitin E3 ligases TRAF2, TRAF3, and TRAF6 to synthesize polyubiquitin chains via the C‐terminal TIMs of MAVS [68]. On the one hand, TRAF family member‐associated NF‐κB activator (TANK) interacts with TRAF3 and TANK‐binding kinase 1 (TBK1), and NF‐κB Essential Modulator (NEMO) senses K63‐linked polyubiquitination to mediate activation of TBK1‐IKKε, leading to TBK1 and IκB kinase ε (LKKε)‐dependent phosphorylation of IRF3/IRF7 and production of type I and type III IFNs [65, 68]. On the other hand, polyubiquitination of Receptor‐Interacting Protein Kinase 1 (RIPK1) and NEMO is mediated by TRAF6 through K63‐linked ubiquitination, which then activates the IKK complex. This leads to the phosphorylation of Inhibitor phosphorylation of Inhibitor of Nuclear Factor kappa B alpha (IκBα), the activation of NF‐κB and NF‐κB‐induced genes [65]. Afterward, repressed NF‐κB signaling results in the production of antiviral cytokines (Figure 1). The SARS‐CoV‐2 membrane (M) protein interacts with RIG‐I, MAVS, and TBK1 to impede the RIG‐I/MDA‐5‐MAVS signaling pathway, thereby inhibiting the production of type I and Type III IFN, ultimately leading to a weakened immune system against the virus and an enhanced viral replication [69].

As mentioned above, ubiquitination plays an indispensable role in the RIG‐I pathway. Meanwhile, A20, acting as a ubiquitin editing enzyme, has the capability to impair the IFN response that relies on RIG‐I through various mechanisms. First, A20 can remove K63‐linked ubiquitin chains from RIPK1, which depends on its deubiquitinating (DUB) activity of the amino‐terminal ovarian tumor (OTU) domain, and add K48‐linked ubiquitin to RIPK1, which is dependent on its Ub‐binding function of zinc finger (ZnF) domains, thus targeting RIPK1 for proteasomal degradation, ultimately preventing the activation of NF‐κB and protecting the cells [70, 71]. Second, A20 can also inhibit the activity of the IKK complex by being recruited to the C‐terminus of NEMO, thereby destabilizing the NF‐κB activation pathway [72]. Additionally, A20 can interact with TBK1 and IKKε, thereby inhibiting their activity and blocking the phosphorylation and subsequent dimerization of IRF3, relying on its ZnF7 domain [65]. Moreover, A20 can impair IRF7 transcriptional activity by negatively regulating K63‐linked polyubiquitination of IRF7 through its N‐terminal OTU domain, particularly during Epstein–Barr Virus (EBV) infection [73]. Otherwise, studies have demonstrated that A20 expression is efficiently induced by the IAV NS1 protein, and while A20 can restrain antiviral defenses as described above, it thereby facilitates IAV replication [34]. In general, this evidence suggests that A20 is a crucial regulator in the dampening antiviral response.

More importantly, there are also clues that inspired us to explore the potentially specific role of A20 in SARS‐CoV‐2 infection. M protein of SARS‐CoV can bind with RIG‐I, TRAF3, TBK1, and IKK and prevent the interaction of TRAF3 with its downstream effectors to dampen signaling, thus restraining IFN production [74]. Meanwhile, TRAF3 is modified with K63‐linked polyubiquitin chains, providing binding sites for other proteins and transducing signaling. This process can be mediated by TRAF3 itself as well as other proteins [75]. Moreover, TRAF3 tends to degrade after its K48‐linked polyubiquitination, a process that can also be mediated by certain ubiquitin ligases. With both DUB and ubiquitinating activities, A20 is a vital protein in the RIG‐I pathway, where it removes K63‐linked ubiquitin and adds K48‐linked ubiquitin, as well as in many other pathways [29, 76] (we will describe it in detail hereinafter). So, it is reasonable to suspect that A20 may synergize with the M protein of SARS‐CoV to impair the activity and stability of TRAF3, thereby limiting RIG‐I‐induced IFN production.

### 3.2 A20 Regulates Antiviral Response Through Its Impact on the STING Pathway

STING is identified as an intracellular sensor protein that mediates host defense by activating the IFN pathway in response to infections with DNA and RNA viruses [77]. STING, which is activated downstream of cyclic GMP‐AMP synthase (cGAS) during DNA virus infections, is well understood and widely accepted. However, the role of STING in RNA virus infections is not yet fully explored. Interestingly, recent studies have demonstrated that STING also participates in resisting enveloped RNA viruses, such as influenza A, by inducing IFN independently of cGAS [78]. First, STING can interact with RIG‐I and MAVS to form a complex, thereby transmitting RIG‐I signaling to the STING‐IFN axis independently of cGAS [79, 80]. Second, the production of IFN and proinflammatory cytokines, such as TNF, which depend on RIG‐I and MAVS, can synergistically upregulate STING expression. Third, this positive feedback mechanism upgrades the production of STING‐dependent IFNs [80]. Ultimately, enveloped RNA viruses, such as IVA, can directly activate STING through a membrane fusion process, but the mechanism of STING activation under these fusion‐stimulated conditions remains unresolved [78, 80]. SARS‐CoV‐2 NSP8 inhibits the formation of the RIG‐I/MDA5‐MAVS complex; it also directly interacts with TRIF and STING to inhibit subsequent signaling transduction. These result in the inhibition of type I and III IFN responses [49].

In response to upstream signaling, STING dimerizes, migrates to the perinuclear region, recruits TBK‐1, and initiates a phosphorylation cascade that activates both STING and IRF‐3, culminating in the production of IFNs [81]. However, the activity and stability of STING depend on various ubiquitin modifications. For example, some E3 ubiquitin ligases facilitate K63‐linked ubiquitination of STING during viral infections, which then promotes its dimerization, while K48‐linked ubiquitination, mediated by certain E3 ubiquitin ligases, triggers proteasome‐dependent degradation of STING [36, 82]. A20, as a ubiquitin editing enzyme, is capable of replacing K63 ubiquitin chains with K48 ubiquitin chains, thereby promoting the degradation of targeted proteins [70, 71]. These proofs suggest that A20 may be an important protein that targets STING and mediates its degradation through ubiquitin editing. Furthermore, A20's potential role in regulating STING also blocks the activation of IRF3, thereby influencing the subsequent production of IFN.

### 3.3 A20 Regulates Antiviral Response Through Dampen CTL Responses in Bronchial Epithelial Cells

Unlike its function in various other cell types, A20 knockout in airway epithelial cells (A20^AEC‐KO^) in mice did not change the type I IFN response or viral clearance. However, A20 deficiency in club cells of the bronchial epithelium improved disease outcomes during IAV infection, apparently by increasing tolerance. Jonathan Maelfait et al. showed that the absence of A20 in club cells resulted in attenuated pulmonary CTL in response to IAV during the later stage of infection (about 8 days after the infection). This was attributed to reduced levels of the monocyte‐recruiting chemokine C‐C motif chemokine ligand 2 (CCL2), which were elevated in A20^WT^. Because the recruitment of monocytes and CD11b^+^ macrophages to the lungs would decrease after CLL2 reduction [35]. Another study also showed that airway epithelial cells are the primary producers of CCL2 during IAV infection and that the expression of CCL2 in myeloid cells is not affected by the deficiency of A20. However, this finding further demonstrates that the absence of A20 in airway epithelial cells plays a specific role in tolerating IAV and alleviates tissue damage [82]. However, these results suggest an inverse function of A20 in negatively regulating CCL2, and the precise mechanism by which tolerance to IAV is increased following A20 knockout in club cells remains unclear.

p21‐activated kinase 1 (PAK1) plays a vital role in regulating the cytoskeleton and the inflammatory response, and research has indicated its involvement in lung inflammation triggered by coronaviruses [83]. After entering host cells through the interaction of its S protein with the ACE2, SARS‐CoV‐2 causes a downregulation of ACE2 expression in the host. This downregulation subsequently activates PKA1 and CCL2, leading to viral dissemination, immune dysregulation, and excessive inflammatory responses. In this context, A20 demonstrates a negative regulatory function with respect to CCL2. We hypothesize that the modulation of A20 may play a significant role in inhibiting the inflammatory responses induced by COVID‐19 [83, 84, 85]. In addition, research on SARS‐CoV and CCL2 suggests that a mechanism of immune evasion by SARS‐CoV might involve low levels of antiviral cytokine response and intense chemokine upregulation [86], while A20 deficiency in airway epithelial cells rectified the background of elevated CCL2. So, A20 in airway epithelial cells might be a potential target to improve the outcome of patients with SRAS‐CoV‐2 infection.

## 4 Anti‐Inflammatory Function of A20

### 4.1 A20 Restricts TNF by Inhibiting the NF‐κB Signaling Pathway

Studies have shown that the N protein of SARS‐CoV‐2 specifically induces NF‐ κB‐mediated inflammatory responses in renal epithelial cells (RECs) [87]. NF‐κB, which can be activated by Lipopolysaccharide (LPS), TNF, IL‐1, IL‐17, and so on, is a well‐known signaling pathway that plays an important role in the induction of many cytokines involved in inflammation, such as IL‐1, IL‐6, and TNF [88]. Elevated levels of these cytokines are closely associated with severe COVID‐19 infection and the development of ARDS [47, 89]. Therefore, inhibiting NF‐κB activation is recognized as a potentially promising therapeutic approach for severe COVID‐19 patients due to its ability to reduce proinflammatory cytokines [90]. A20 can limit the activation of NF‐κB in multiple pathways, mainly through its two ubiquitin‐editing domains.

First, in the TNF‐induced NF‐κB pathway, A20 can inhibit the modification of NEMO through its carboxy‐terminal domain, composed of seven C2/C2 ZnFs functioning as a ubiquitin ligase thereby depressing the activation of the IKK complex (composed of NEMO, IKKα, and IKKβ), which results in the downregulation of NF‐κB activation [91]. Additionally, by polyubiquitinating receptor‐interacting protein (RIP) with K48‐linked ubiquitin chains by means of its ZnF region, A20 thereby targets RIP for proteasomal degradation, leading to dissociation of a TNF‐receptor‐bound primary complex (complex I), which includes RIPK1, from the membrane and eventually downregulating the activation of NF‐κB [71]. In addition to this, A20, through its DUB enzyme from the OTU family [92] can also remove K63‐linked ubiquitin chains from RIPK, which has emerged as the direct substrate of IKKα/IKKβ and plays a major role as a signaling hub downstream of several immune receptors [93], leading to a critical brake in the proximal TNF Receptor 1 (TNFR1) pathway [94] and ultimately attenuating the activation of downstream signaling [71, 72]. Moreover, in the TLR‐induced NF‐κB pathway (due to LPS and IL‐1β stimulation), A20 can remove K63 ubiquitin chains on TRAF6 and then impair IKK complex activity to block the activation of NF‐κB [70]. A20 also downregulates T‐cell receptor (TCR)‐induced NF‐κB activation by removing K63‐linked ubiquitin chains from Mucosa‐associated lymphoid tissue lymphoma translocation protein 1 (MALT1), leading to the prevention of the interaction between MALT1 and the IKK complex [95]. Furthermore, when muramyl dipeptide (MDP) stimulates A20‐deficient cells, increased RIPK2 ubiquitylation and prolonged NF‐κB signaling occurred, and it prompted that A20 also restricts nucleotide‐binding oligomerization domain‐containing protein 2 (NOD2)‐induced NF‐κB signaling [96].

### 4.2 A20 Restricts IL‐6 and IL‐17 Through the Mitogen‐Activated Protein Kinase (MAPK) Signaling Pathway

The MAPK signaling pathway is located upstream of the NF‐κB pathway, reminding us that A20 has multiple anti‐inflammatory effects. After being stimulated by various environmental stresses and inflammatory cytokines, p38, as a conventional MAPK signaling pathway in the extracellular space, phosphorylates MAPK‐activated protein kinases (MK) such as MK2, MK3, and MK5 located in the cytoplasm [97, 98]. This leads to an increase in TNF‐α and IL‐6, which can be attributed to the promotion of translation and stability of their mRNAs [99]. IL‐6 is a critical proinflammatory factor that causes cytokine storms in COVID‐19 [58, 90]. It is also upregulated to recruit neutrophils through enzymatic activation, subsequently activating the NF‐κB signaling pathway in inflammatory diseases [100]. This leads to the formation of neutrophil extracellular traps (NETs), which exacerbate pulmonary inflammation and impair the epithelial–endothelial barrier [101]. In an inflammatory environment, A20 can negatively regulate the increase of IL‐6 [102].

Additionally, A20 eliminates K63 ubiquitination of TRAF6, which is a key event in the downstream inactivation of the NF‐κB and MAPK signaling pathways, which underlies the decreased production of proinflammatory cytokine IL‐17 and subsequently attenuates inflammatory response [103]. Besides, consistent with another research, it showed that the suppression of NF‐κB and MAPK enhanced by A20 resulted in the disaggregation of K63 from NEMO and TRAF6, leading to the reduction of the downstream production of IL‐6 [104]. Meanwhile, as a member of the MAPK family, c‐Jun N‐terminal kinase (JNK) may play a significant role in promoting cell death and triggering inflammatory responses during SARS‐CoV‐2 infection [14, 105]. Surprisingly, overexpressed A20 inhibited the activation of extracellular signal‐regulated kinase (ERK), JNK, and P38 MAPK [106].

### 4.3 A20 Restricts IL‐1 Through Inhibiting Macrophage Necroptosis

As a regulated necrotic cell death modality in a caspase‐independent fashion [107], necroptosis is not only regulated by intramolecular signals but also mediated by the RIPK1–RIPK3 complex, which cooperates with Mixed Lineage Kinase Domain‐Like (MLKL) [108]. Although the specific mechanism remains unclear, interestingly, A20 indirectly affects macrophage necroptosis via reducing phosphorylation of RIPK3 to inhibit the formation of the RIPK1–RIPK3 complex, functioning as an inhibitor of necroptosis [109, 110]. Moreover, the dying cells can release danger‐associated molecular patterns (DAMPs), especially IL‐1 [111]. It has been demonstrated that not only anti‐IL‐6 but also anti‐IL‐1 treatments have yielded good outcomes in severe cases probably by controlling the cytokine storm [112].

It has been verified that when abnormal mutations occur in the ZnF7 domain of A20, the A20^mZnF7/mZnF7^ phenotype is associated with higher serum concentrations of TNF and IL‐6, leading to macrophage necroptosis and inflammation. This is due to the degradation of methionine 1 (M1)‐linked ubiquitin chains connecting TNFR1 signal complexes by DUBs, which indirectly indicates that A20 stabilizes the complexes through its ZnF7 domain under normal circumstances and eventually relieves inflammation [113]. In addition, the assembly of the death‐inducing signaling complex (DISC), which serves as a platform for the activation of initiator caspases in extrinsic apoptosis [114], is accomplished through two different types of homotypic interactions. One of these interactions requires the ubiquitylation of Caspase‐8 to facilitate the activation of Cullin 3 (CUL3). A20, with its deubiquitylating activity, removes ubiquitin from Caspase‐8 and recruits RIPK1 into the DISC, thereby preventing the assembly of the DISC and inhibiting the release of inflammatory cytokines, particularly IL‐1 [26].

### 4.4 A20 Restricts IL‐1 and IL‐18 Through Dampening NLRP3 Activity

Pyrin domain containing 3 (NLRP3) is an inflammatory sensor protein that plays an important role in both inflammatory response and immune regulation. In response to cellular oxidative stress, bacterial infections, and intracellular metabolite changes, the NLRP3 inflammasome is activated, triggering a series of inflammatory responses, the most important of which is the processing and release of the proinflammatory cytokines IL‐1β and IL‐18 [115, 116]. The NLRP3 inflammasome works like a double‐edged sword. On the one hand, the NLRP3 inflammasome can affect the immune response and help the body fight against viruses [116]. On the other hand, when the NLRP3 inflammasome is excessively activated, it triggers cytokine storms, leading to the release of a large number of proinflammatory cytokines and a strong inflammatory response that damages tissues and organs in the body [116]. Activation of NLRP3 inflammasome has been detected in monocytes and macrophages of COVID‐19 patients [117]. Moreover, the elevated levels of IL‐1β and IL‐18 in the serum of patients with COVID‐19 were positively correlated with the severity of the disease [118]. Excessive release of IL‐18 and IL‐1β in severe COVID‐19 cases leads to cytokine storms and severe tissue damage. It has been confirmed that the S protein, E protein, and N protein of SARS‐CoV‐2 are closely related to the activation of the NLRP3 inflammasome [21, 119, 120]. For example, the N protein of SARS‐CoV‐2 promotes the interaction between NLRP3 and ASC, leading to the formation of the N‐NLRP3‐ASC complex, which triggers the activation of the NLRP3 inflammasome [21]. The S protein of novel coronavirus binds to ACE2 and also promotes the activation of the NLRP3 inflammasome [120].

It has been revealed that there is a close association between the NLRP3 inflammasome and A20. The deficiency of A20 in various types of cells, including myeloid cells, microglial cells, and macrophages, activates the NLRP3 inflammasome and leads to the release of mature IL‐1β [23, 113, 121, 122]. It has been verified that A20 alleviates inflammation by restricting the activation of NF‐κB to suppress the production of NLRP3 priming, a process that depends on NF‐κB [123]. But interestingly, the study in murine A20‐deficient macrophages has revealed an increase in basal NLRP3 levels but also in TLR‐induced NLRP3, thereby potentially providing a clue to the existence of additional mechanisms to regulate NLRP3 activity [113]. Besides, A20 can attenuate inflammatory activity by dampening K63‐ubiquitylation of pro‐IL‐1 beta (pro‐IL‐1β), which underlyingly activates Casp1 to weaken pyroptosis and decrease the production of IL‐1β [23, 121]. Furthermore, ZnF4 and ZnF7 motifs of A20 can synergistically downregulate NLRP3 inflammasome activity directly [124]. A20 can also regulate necroptosis‐induced NLRP3 inflammasome activation as described above.

## 5 A20 Expression Correlates With the Infectious Stage of SARS‐CoV‐2

In addition to the A20's indispensable role in mediating the pathological processes of viral infection and inflammation, we also demonstrate that A20 has complex regulatory relationships with numerous cytokines that are abnormally activated during SARS‐CoV‐2 infection. This suggests a potential association between A20 and COVID‐19. To further investigate, we have compiled clinical cases for an in‐depth study of COVID‐19.

**Figure 2:**
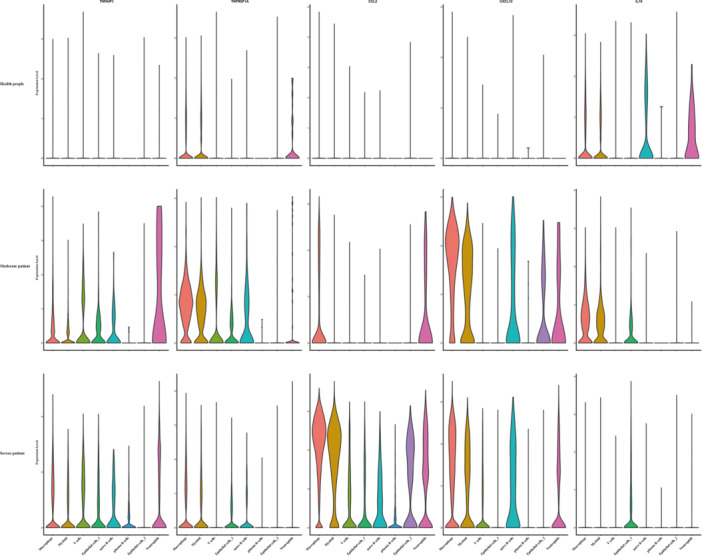
Expression of various molecules differs across the varying severity of SARS‐CoV‐2 infection. In healthy individuals, A20 was minimally expressed, and IL‐18 was present in small amounts in normal cells. In moderate patients, the expression of A20 was significantly upregulated in all immune cells except for plasma B cells and epithelial cells. TNFSF1A was notably increased in macrophages, myeloid cells, T cells, epithelial cells, and naive B cells. CCL2 was enhanced primarily in macrophages and neutrophils, though not significantly. CXCL10 was substantially activated and expressed, particularly in macrophages and myeloid cells. The expression of IL‐18 was upregulated in macrophages and myeloid cells but suppressed in naive B cells and neutrophils. In severe patients, A20 expression was significantly reduced in neutrophils compared to moderate patients. TNFRSF1A was suppressed in almost all kinds of immune cells, while CCL2 was essentially stimulated in multifarious cells, especially in macrophages and myeloid cells. The expression of CXCL10 was slightly decreased, but the overall trend was not distinct. IL‐18 was significantly attenuated with only a small amount expressed in epithelial cells.

We downloaded the single‐cell RNA‐seq data set from the NCBI GEO database, which includes data from healthy individuals and patients with varying severity of COVID‐19 disease (accession number GSE145926) [125]. Disease severity was defined as moderate, severe, and critical, according to the “Diagnosis and Treatment Protocol of COVID‐19 (the 7th Tentative Version)” by the National Health Commission of China issued on 3 March 2020 (http://www.nhc.gov.cn/yzygj/s7653p/202003/46c9294a7dfe4cef80dc7f5912eb1989.shtml). The quality control of the single‐cell RNA‐seq data set was performed with the following criteria: gene number between 250 and 4000, Unique Molecular Identifiers (UMI) count > 1000, and mitochondrial gene percentage < 0.1. The matrices of all samples were integrated using Seurat v.3.2.3 to remove batch effects across different donors [126]. In the parameter settings, the first 30 dimensions from canonical correlation analysis (CCA) and principal component analysis (PCA) were utilized. Violin plots were applied to depict molecules with significantly different expression levels across varying severities of SARS‐CoV‐2 infection (Figure 2).

In healthy controls, A20 remained virtually undetectable, but IL‐18 was expressed in small amounts in macrophages, myeloid cells, naive B cells, and neutrophils. The expression of each cytokine was significantly higher in patients with mild infections. Compared with healthy controls, the expression of A20 was significantly upregulated in all immune cells, especially neutrophils but remained at low levels in plasma B cells and epithelial cells. TNF Receptor Superfamily Member 1 A (TNFSRF1A) was considerably increased in macrophages and myeloid cells and was also upregulated in T cells, epithelial cells, and naive B cells. CCL2 expression was mainly enhanced in macrophages and neutrophils but not significantly. C‐X‐C motif chemokine ligand 10 (CXCL10) was substantially activated and expressed in several immune cells, particularly in macrophages and myeloid cells. The expression of IL‐18 was upregulated in macrophages and myeloid cells, but interestingly, it was greatly suppressed in naive B cells and neutrophils. In patients with severe infections, A20 expression in immune cells was similar to that observed in mild infections but was significantly reduced in neutrophils. TNFRSF1A expression was suppressed in almost all types of immune cells, while CCL2 was essentially stimulated in various cells, particularly in macrophages and myeloid cells. The expression of CXCL10 was slightly decreased, but the overall trend was not distinct. IL‐18 expression was also significantly attenuated, with only a small amount expressed in epithelial cells.

These phenomena suggest that multiple cytokines are involved in immune regulation and inflammatory processes, which ultimately contribute to the underlying pathology of disease development in viral infections. Although it is well‐established that these cytokines are significantly activated and participate in cytokine storms, they engage in viral infections and immune responses in pleiotropic ways. IL‐18 is highly expressed in the pulmonary macrophages of COVID‐19 patients [127], while a positive correlation with poor clinical outcomes or side effects has been widely demonstrated [118, 128, 129, 130]. The expression of TNFRSF1A also significantly elevates, but a distinct difference lies in its levels among patients with different severities of symptoms after viral infection [131, 132, 133]. CCL2 has been identified as a biomarker predicting the severity of COVID‐19 in serum and plasma [134, 135], correlating with poor prognosis, and even mortality, especially in moderate to severe cases [136, 137]. CXCL10, one of the topmost prioritized anti‐inflammatory targets and the highest priority drug targets, is engaged in olfactory dysfunction in COVID‐19 infections [138].

In addition to their close relationship with COVID‐19, these inflammatory cytokines may have potential links to A20 through various mechanisms. In a mouse myocarditis model, a downregulation of A20 was observed simultaneously with an overexpression of IL‐18. Furthermore, a close relationship between IL‐18 and the dysfunction of the A20/NLRP3 inflammasome axis has been observed in various diseases, such as Parkinson's disease [139] and diabetes [140]. Numerous studies have demonstrated that A20 is recruited to the TNFRSF1A complex I within minutes of sensing TNF [113, 141, 142], ultimately downregulating the NF‐κB signaling pathway and alleviating cell apoptosis [143]. The inhibitory effect of A20 on CCL2 has been observed in multiple diseases, such as vitiligo [144], systemic lupus erythematosus (SLE) [145], and acute endoplasmic reticulum (ER) stress [146], and these studies all point to the fact that A20 downregulates CCL2 primarily by inhibiting the NF‐κB signaling pathway. Furthermore, CXCL10 plays multiple roles, being strongly induced by IFN‐α, ‐β, and ‐γ [147]. Its biological actions are mainly dependent on the JAK/STAT1/IRF1 signal transduction pathway [148], the NF‐κB pathway [149], or the p38 signaling pathway [150]. Intriguingly, A20 targets all three of these typical inflammation‐associated signaling pathways, reminding us of the potential connection between A20 and CXCL10.

## 6 Conclusion

In summary, A20, as a direct inhibitory protein of multiple inflammatory and apoptosis‐related signaling pathways, might be one of the most critical anti‐infectious and anti‐inflammatory factors involved in the pathogenesis of COVID‐19. It effectively suppresses the immune damage and inflammatory storm caused by viral infection. However, although we hypothesize that A20 could be a promising clinical therapeutic target for COVID‐19, treatments capable of precisely controlling A20 expression to induce antiviral activities are currently lacking. Thus, intensive research on A20, along with potential therapeutic treatments, should be further deeply pursued.

## Author Contributions

Qing Zhao and Bin Shao designed the study and reviewed the manuscript. Jiuxuan Li reviewed the manuscript. All authors read and approved the final manuscript.

## Ethics Statement

The authors have nothing to report.

## Consent

The authors have nothing to report.

## Conflicts of Interest

The authors declare no conflicts of interest.

## Data Availability

All data used to support the findings of this study are included in the article. (This work has not been published or submitted for publication elsewhere, either completely or in part, or in another form or language. These two figures are original works by the author and do not have any copyright issues.)
